# Transcriptional alterations in SKAP1-associated inside-out signaling in CNS-infiltrated pediatric T-cell acute lymphoblastic leukemia

**DOI:** 10.1038/s41420-026-03452-w

**Published:** 2026-11-05

**Authors:** Sabina Enlund, Konstantinos Papadakis, Jacob Short, Ola Hermanson, Anna Nilsson, Qingfei Jiang, Frida Holm

**Affiliations:** 1 Department of Women’s and Children’s Health, Division of Pediatric Oncology and Surgery, Karolinska Institutet, Stockholm, Sweden; 2 Department of Oncology-Pathology, Karolinska Institutet, Stockholm, Sweden; 3 Department of Neuroscience, Karolinska Institutet, Stockholm, Sweden; 4 Division of Regenerative Medicine, Department of Medicine, Moores Cancer Center, University of California, San Diego, La Jolla, CA USA

**Keywords:** Acute lymphocytic leukaemia, Transcriptional regulatory elements

## Abstract

A major obstacle to improving treatment efficacy and long-term survival in children with T-cell acute lymphoblastic leukemia (T-ALL) is the limited understanding of the molecular features associated with central nervous system (CNS) infiltration. Leukemia cells that migrate to the CNS can evade systemic therapy, contributing to disease progression and relapse. A better understanding of the transcriptomic profiles associated with CNS infiltration may provide insights into the underlying molecular programs involved in leukemic cell migration. Therefore, we aimed to identify transcriptional alterations that characterize CNS-infiltrated pediatric T-ALL patients. RNA-sequencing analysis revealed enrichment of genes associated with T-cell receptor (TCR) signaling, including SKAP1, a key adapter protein in the TCR-mediated inside-out signaling pathway, in both CNS-infiltrated and CNS-relapsed pediatric T-ALL. While inside-out signaling regulates normal T-cell adhesion and migration, its role in malignant leukemic cell migration remains unclear. In addition, T-ALL cells exposed to methotrexate and co-cultured with meningeal cells exhibited alterations in genes downstream of the TCR. Knockdown of SKAP1 resulted in reduced viability and proliferation of T-ALL cells. Collectively, these findings define a transcriptional profile associated with CNS infiltration in pediatric T-ALL, including altered TCR signaling and SKAP1 expression. Whether these alterations directly contribute to leukemic cell migration into the CNS remains to be established.

**Figure:**
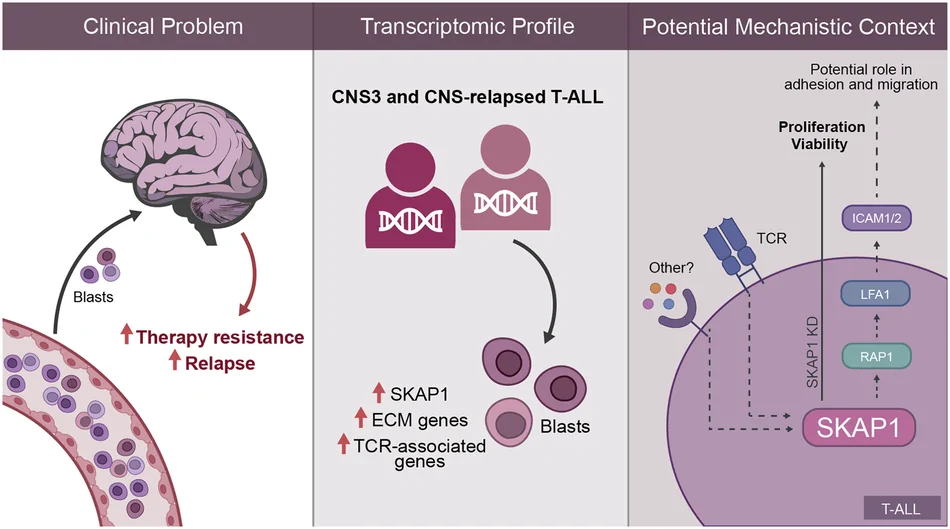

## Introduction

T-cell acute lymphoblastic leukemia (T-ALL) is an aggressive hematological malignancy characterized by aberrant proliferation of immature lymphocytes [1]. The pathology of T-ALL is complex, largely due to the heterogeneity of patient genetics and chromosomal abnormalities [2]. Over the past decades, the 5-year overall survival for children diagnosed with T-ALL has increased immensely, reaching ~90% [2, 3]. However, a substantial risk of relapse remains, partly due to migrating leukemia cells that enter and reside in the central nervous system (CNS). Leukemia cells that infiltrate the CNS can evade systemic chemotherapy, leading to the implementation of intrathecal methotrexate (MTX) in routine care [4, 5]. CNS-directed therapy is administered to all children with T-ALL, which has greatly improved the survival outcomes, but can cause severe neurotoxicity and late effects [5–7]. In order to enhance current treatment options and minimize adverse effects, we need to advance our understanding of how leukemia cells infiltrate and survive in the CNS [4].

The main site of leukemia infiltration is the meninges—the protective layers that surround the brain and spinal cord—which contain the cerebrospinal fluid (CSF) [5, 8]. Potential entry routes for leukemia cells into the CNS include the blood-leptomeningeal barrier and the blood–CSF barrier [5]. Normal T-cells can migrate using a range of different mechanisms, including chemokine gradients, cytoskeletal remodeling, and integrin-mediated adhesion [9–12]. LFA1 is a key integrin involved in T-cell adhesion and migration through binding to the adhesion molecule ICAM1. Activation of LFA1 can be induced through a pathway called inside-out signaling, initiated primarily by the T-cell receptor (TCR) [11, 12]. This signaling pathway involves adapter proteins, including SKAP1 (also known as SKAP55), which links TCR signaling to downstream activation of RAP1 and subsequent LFA1 activation [13, 14]. While the inside-out signaling pathway is well characterized in normal T-cells, its regulation and potential role in malignant T-cell migration and CNS infiltration remain unclear.

A better understanding of the transcriptional profiles associated with CNS infiltration in T-ALL may provide insights into the underlying molecular programs involved in leukemic cell migration. We therefore aimed to investigate transcriptional features associated with CNS infiltration by analyzing RNA-sequencing data from pediatric T-ALL patients (publicly available by the TARGET initiative). Enrichment analysis of T-ALL samples classified as CNS-infiltrated or CNS-relapsed compared with CNS-negative samples revealed altered expression of genes and pathways associated with TCR signaling across both comparisons. Among these, adapter protein SKAP1 emerged as a prominent feature associated with CNS-infiltrated and CNS-relapsed T-ALL. Furthermore, T-ALL cells were co-cultured with CNS-derived meningeal cells and exposed to MTX, resulting in alterations in genes involved in the inside-out signaling pathway. Knockdown of SKAP1 in T-ALL cells leads to reduced cell proliferation and viability. Together, these findings identify transcriptional characteristics associated with CNS infiltration and CNS relapse in pediatric T-ALL, including altered inside-out signaling and SKAP1 expression. Whether these alterations directly contribute to CNS infiltration remains to be established.

## Results

### CNS-infiltrated and CNS-relapsed T-ALL exhibit transcriptional alterations in ECM organization and SKAP1 expression

**Fig. 1:**
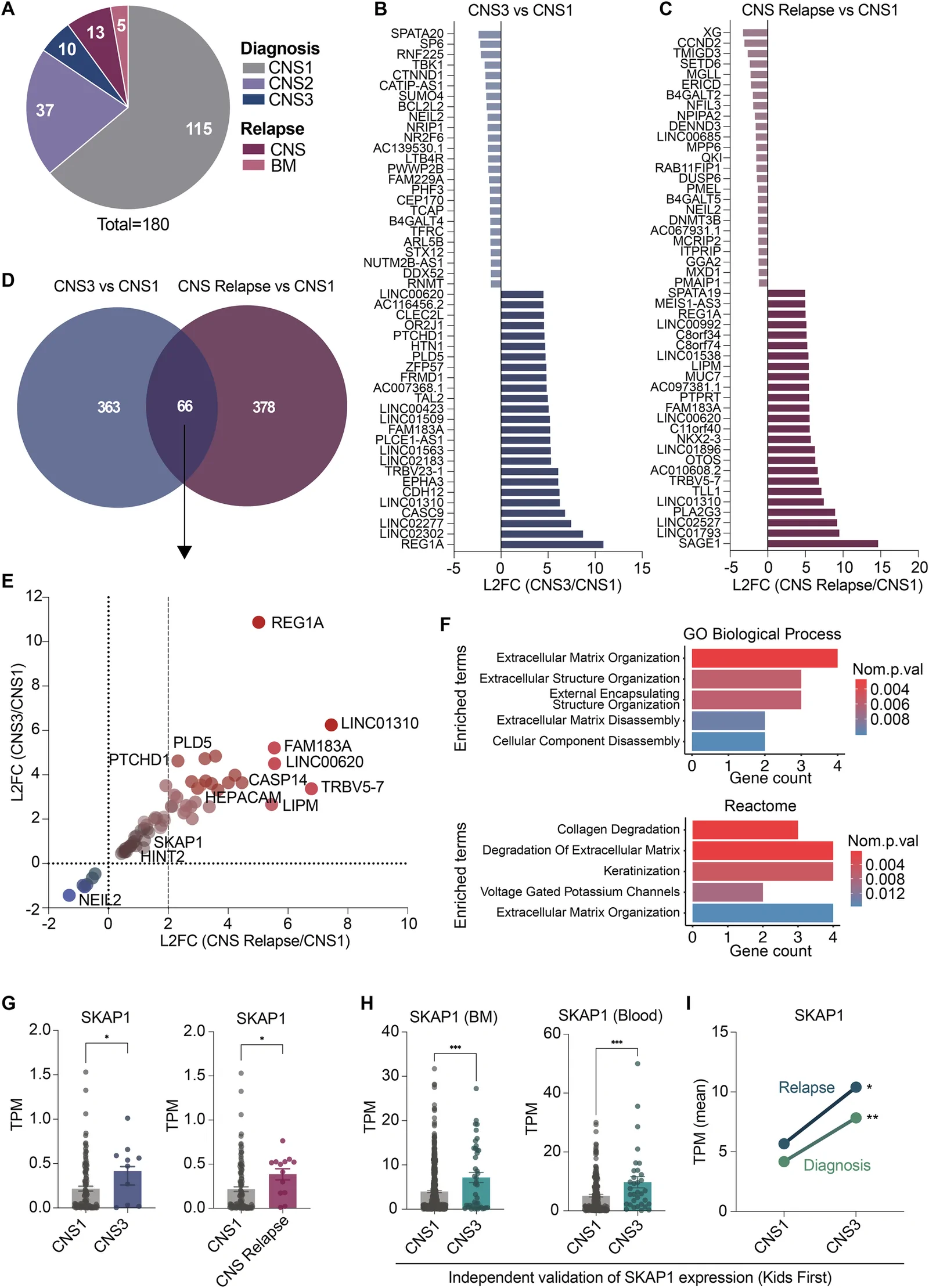
Differential gene expression in CNS-infiltrated and CNS-relapsed T-ALL compared with CNS-negative T-ALL. **A** Clinical characteristics of included pediatric T-ALL samples (total *n* = 180). Samples from the time of diagnosis were subdivided based on the clinical characterization of CNS infiltration, into CNS1 (*n* = 115), CNS2 (*n* = 37), or CNS3 (*n* = 10). Samples taken at relapse were subdivided by site of relapse: CNS (*n* = 13) or BM (*n* = 5). **B** Top differentially expressed genes (*P* < 0.05) in CNS3 (*n* = 10) samples compared to CNS1 (*n* = 115) samples. Data displayed as L2FC. Significance calculated by unpaired Students *t* test. **C** Top differentially expressed genes (*P* < 0.05) in CNS relapse (*n* = 13) samples compared to CNS1 (*n* = 115) samples. Data displayed as L2FC. Significance calculated by two-tailed unpaired Students *t* test. **D** Venn diagram of significantly differentially expressed genes (*P* < 0.05) in CNS3 (*n* = 10) and CNS relapse (*n* = 13) compared to CNS1 (*n* = 115). Significance calculated by two-tailed unpaired Students *t* test. **E** Volcano plot of all common differentially expressed genes (*P* < 0.05, total of 66 genes) in CNS3 (*n* = 10) and CNS relapse (*n* = 13) compared to CNS1 (*n* = 115). Data displayed as L2FC in CNS3 (*y* axis) and CNS relapse (*x* axis) compared to CNS1. Significance calculated by two-tailed unpaired Students *t* test. **F** Enrichment analysis of the commonly differentially expressed genes (total of 66 genes) in CNS3 (*n* = 10) and CNS relapse (*n* = 13) compared to CNS1 (*n* = 115) using EnrichR (v3.2) in R (version 4.3.1), with GO biological processes (upper graph) and Reactome (lower graph) gene sets. Significance is displayed as a nominal *P* value. **G** Expression of SKAP1 in CNS3 (*n* = 10) compared to CNS1 (*n* = 115) samples (left graph) and CNS relapse (*n* = 13) compared to CNS1 (*n* = 115) samples (right graph). Data displayed as TPM, mean ± SEM. Significance calculated by two-tailed unpaired Students *t* test (*P < 0.05). **H** Expression of SKAP1 in CNS3 compared to CNS1 samples in an independent T-ALL cohort (Kids First), in samples from both BM (CNS1 *n* = 516, CNS3 *n* = 42, left graph and blood (CNS1 *n* = 208, CNS3 *n* = 33, right graph). Data displayed as TPM, mean ± SEM. Significance calculated by two-tailed unpaired Students *t* test (***P < 0.001). **I** Mean expression of SKAP1 displayed as TPM in CNS3 compared to CNS1 samples in diagnosis (CNS1 *n* = 654, CNS3 *n* = 62) and relapse (CNS1 *n* = 70, CNS3 *n* = 13) separately, from the Kids First dataset. Significance calculated by two-tailed unpaired Students *t* test (*P < 0.05, **P < 0.01).

To identify transcriptional alterations that characterize CNS-infiltrated and CNS-relapsed T-ALL samples, RNA-sequencing analysis was performed on 180 pediatric bone marrow (BM)-derived T-ALL samples, collected at diagnosis and relapse (publicly available by the TARGET initiative, non-longitudinal) (Supplementary Table S1). BM samples were used as an accessible source to identify molecular features associated with CNS disease, given the limited availability and invasive nature of CSF samples in pediatric patients. Diagnosis samples were classified as CNS1, CNS2, or CNS3, while relapse samples were grouped based on the site of relapse, as CNS or BM (Figs. 1A and SF1A) [15]. We focused on CNS3 samples (*n* = 10) and CNS-relapsed samples (*n* = 13), compared with CNS1 samples (*n* = 115). Although most genes showed lower expression in CNS3 and CNS-relapsed samples compared with CNS1 samples, most significantly differentially expressed genes were upregulated in both CNS3 (74%) and CNS relapse (85%) (Fig. SF1B, C). Among the top altered genes, TAL2, a transcription factor closely related to T-ALL oncogene TAL1 was upregulated in CNS3 patients, and Cyclin D2 (CCND2), a known cell cycle regulator, was downregulated in CNS relapse patients (Fig. 1B, C) [16–18].

In both CNS3 and CNS relapse samples, more than 400 genes were differentially expressed compared with CNS1 samples, with 66 genes commonly dysregulated between both groups (Figs. 1D, E and SF1D). Among these genes, we identified an upregulation of HINT2, which controls mitochondria-induced apoptosis, as well as HEPACAM, an adhesion molecule linked to cell cycle arrest in breast cancer cells (Figs. 1E and SF1D) [19, 20]. The commonly dysregulated genes were analyzed using EnrichR [21], which revealed an association with extracellular matrix (ECM) organization and degradation in both the Reactome and Gene Ontology (GO) libraries (Fig. 1F). Genes involved in these pathways included COL9A3, TMPRSS6 and metalloproteinase MMP11, which were all upregulated in CNS3 and CNS relapse compared with CNS1 samples (Fig. SF1E, F). Notably, adapter protein SKAP1 was among the 66 commonly altered genes and was upregulated in both CNS3 and CNS relapse compared with CNS1 patients (Figs. 1E, 1G). As the TARGET cohort contained relatively few CNS3 samples, we validated this finding in a larger pediatric T-ALL cohort (*n* = 799, from Kids First, accessed through Synapse: syn54032669), including samples taken at the time of diagnosis or relapse (Fig. SF1G) [22]. In samples derived from both BM and blood, SKAP1 expression was higher in CNS3 compared with CNS1 patients (Fig. 1H). Furthermore, this difference was observed in both diagnostic and relapse samples separately (Figs. 1I and SF1H). Together, these findings identify SKAP1 as a transcriptional feature associated with CNS disease across two independent pediatric T-ALL cohorts.

### Alterations to the TCR complex and SKAP1 in CNS-infiltrated and CNS-relapsed T-ALL

**Fig. 2:**
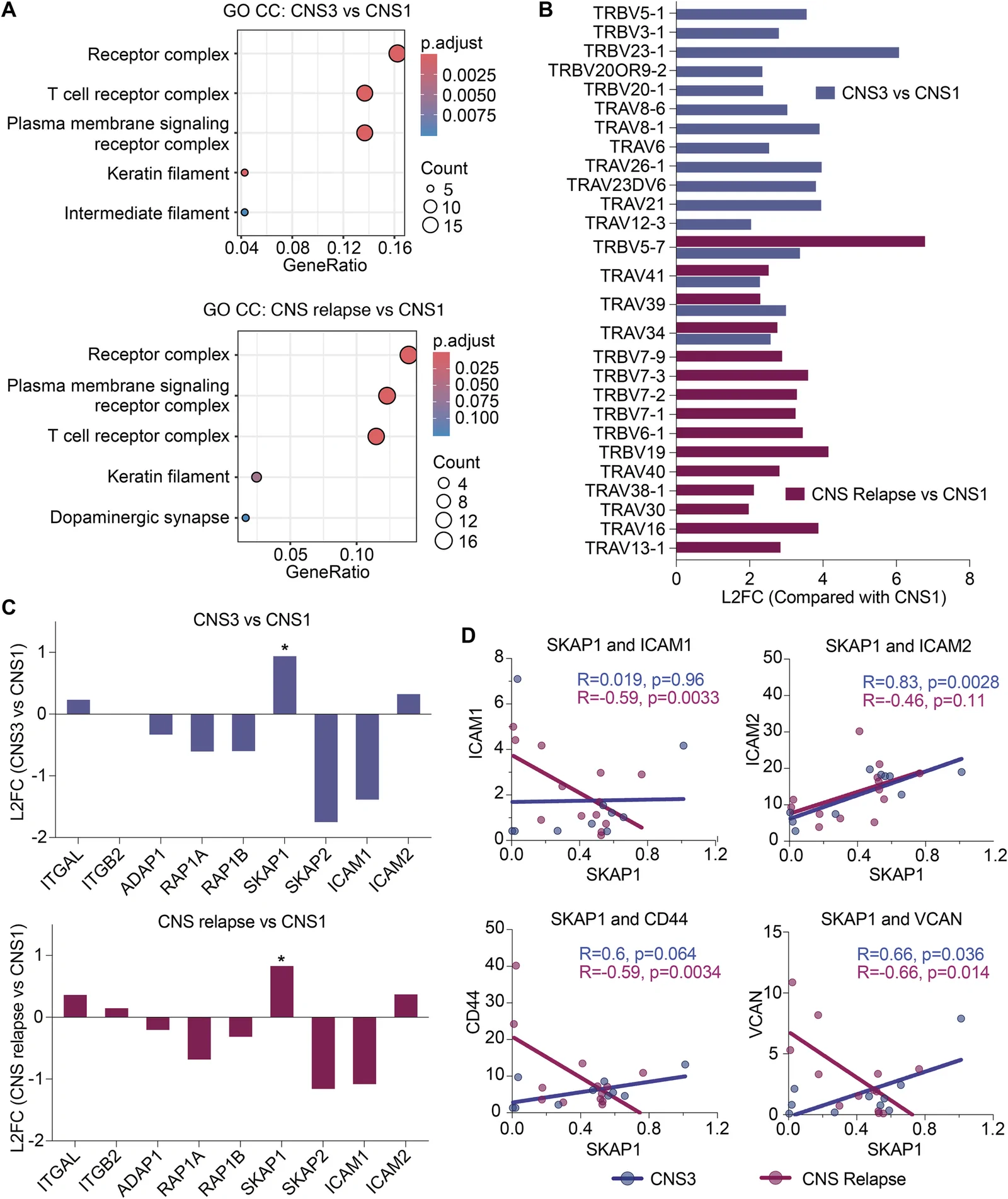
Dysregulation of the TCR complex and SKAP1 in CNS-infiltrated and CNS-relapsed T-ALL. **A** ORA of differentially expressed genes (*P* < 0.05, L2FC > 2) in CNS3 (*n* = 10) compared to CNS1 (*n* = 115) samples (upper graph) and CNS relapse (*n* = 13) compared with CNS1 (*n* = 115) samples (lower graph), performed in R (version 4.3.1) using the GO cellular compartment (CC) gene set. Significance displayed as adjusted *P* value. **B** Dysregulated genes (*P* < 0.05) from variable segments of the TCR complex in CNS3 (*n* = 10) and CNS relapse (*n* = 13) compared with CNS1 (*n* = 115). Data displayed as L2FC. Significance calculated by two-tailed unpaired Students *t* test. **C** Expression of ITGAL, ITGB2, ADAP1, RAP1A, RAP1B, SKAP1, SKAP2, ICAM1 and ICAM2 in CNS3 (*n* = 13) compared with CNS1 (*n* = 115) samples in the upper panel and CNS relapse (*n* = 13) compared with CNS1 (*n* = 115) samples in the lower panel. Data displayed as L2FC. Significance calculated by two-tailed unpaired Students *t* test (*P < 0.05). **D)** Correlations of SKAP1 and ICAM1, ICAM2, CD44, and VCAN in CNS3 (*n* = 10) and CNS relapse (*n* = 13) samples separately (CNS3 in blue, CNS relapse in maroon). Correlation was calculated using Pearson correlation coefficients, two-tailed with 95% confidence interval. Data displayed as TPM values (*x* axis and *y* axis) and results shown as the R value and *P* value of each group.

To further characterize CNS-infiltrated T-ALL, over-representation analysis (ORA) was performed using all significantly differentially expressed genes in CNS3 and CNS relapse compared with CNS1, respectively. Analysis of GOs revealed an enrichment of genes in Cellular Compartments (CC) related to the TCR complex in both CNS3 and CNS relapse samples (Figs. 2A and SF2A, B). Many genes coding for variable regions of the α and β chains of the TCR were found to be upregulated in CNS3 and CNS-relapse samples (Fig. 2B). We previously identified SKAP1 as one of the 66 genes commonly altered in both CNS3 and CNS relapse samples (Fig. 1E, G), which is crucial for TCR-mediated inside-out signaling. Notably, other components of inside-out singling, such as ITGAL, ITGB2, ADAP1, RAP1 (RAP1A and RAP1B), and ICAM1, together with SKAP1-paralogue SKAP2, were not significantly altered in CNS3 or CNS relapse patients compared with CNS1 patients (Fig. 2C).

Next, we explored the transcriptional relationships of SKAP1 by examining correlations between SKAP1 expression and selected genes identified in our differential expression analyses, as well as genes known to facilitate migration and adhesion, or are associated with TCR signaling. In CNS3 patients, nearly all substantial correlations (*R* > 0.6 or *R* < −0.6) were positive (96%), while CNS relapse samples showed a larger variety of associations (60% positive, 40% negative) (Fig. SF2C). CNS relapse patients displayed a negative correlation between SKAP1 and adhesion genes ICAM1 and CD44, while CNS3 patients showed no correlation, or a positive correlation (Fig. 2D). Both CNS3 and CNS relapse samples displayed a positive correlation between SKAP1 and ICAM2, which like ICAM1, binds to LFA1, but has a more controversial role in tumor cells (Fig. 2D) [23]. Opposing correlations (positive in CNS3 and negative in CNS relapse) were observed between SKAP1 and VCAN, a gene that is associated with epithelial–mesenchymal transition (EMT) (Fig. 2D) [24]. In both groups, a positive correlation was identified between SKAP1 and NFATC1, a transcription factor downstream of the TCR, as well as between SKAP1 and HINT2, previously identified as upregulated in CNS-associated samples (Fig. SF2D) [19, 25]. Together, these analyses identified both shared and divergent transcriptional relationships between SKAP1 and genes involved in TCR signaling, adhesion, and migration in CNS3 and CNS-relapsed T-ALL.

### CNS-associated transcriptional features included altered integrin signaling and SKAP1 expression

**Fig. 3:**
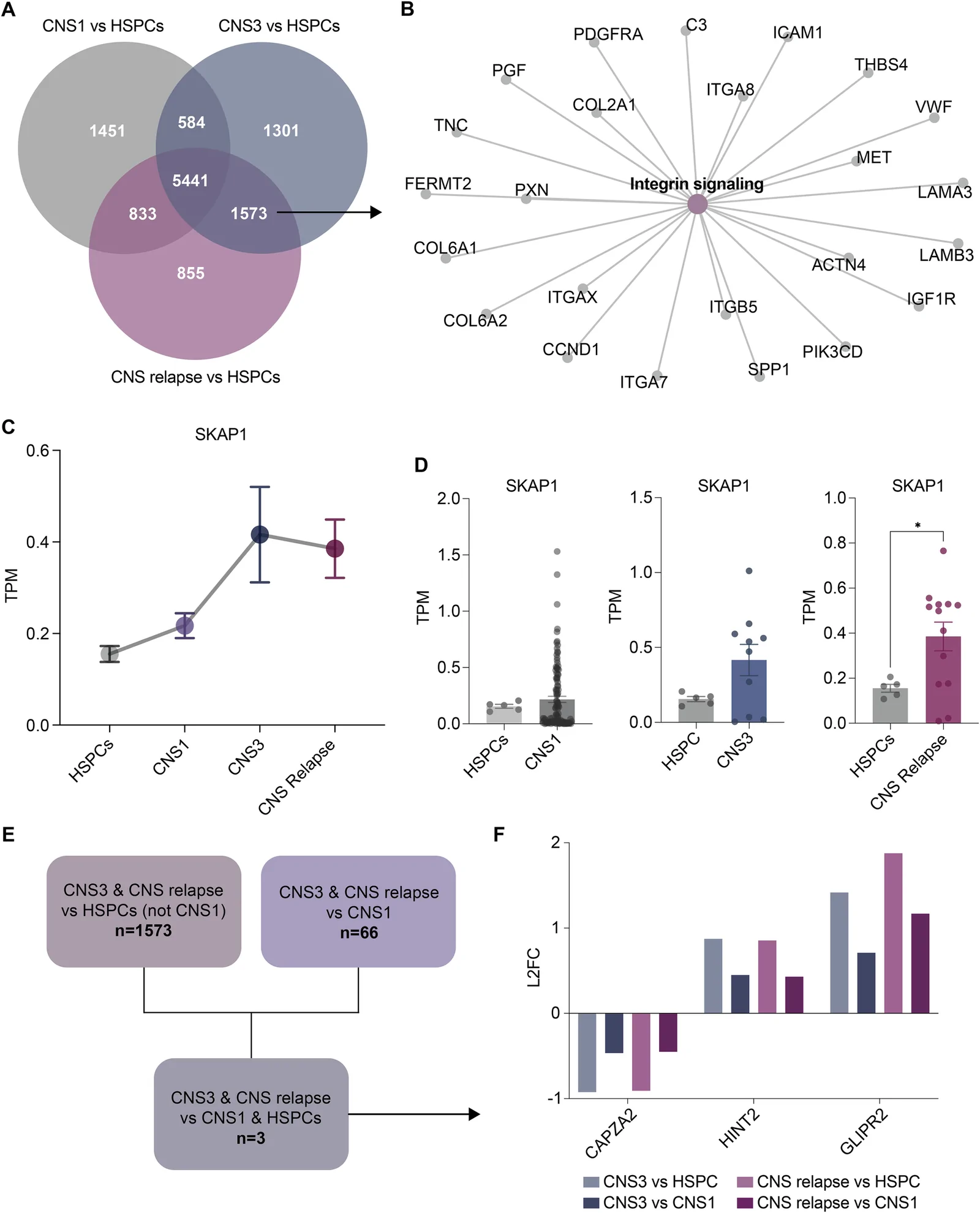
Identification of transcriptional alterations in CNS-associated T-ALL compared with CNS-negative T-ALL and HSPCs. **A** Venn diagram of differentially expressed genes (*P* < 0.05) CNS3 (*n* = 10), CNS relapse (*n* = 13) and CNS1 (*n* = 115) compared with HSPCs (CD34 + CB, *n* = 5). Significance calculated by two-tailed unpaired Students *t* test. **B** Network plot of the KEGG pathway Integrin signaling, showing enriched genes (*P* < 0.05) exclusive to CNS3 (*n* = 10) and CNS relapse (*n* = 13) compared with HSPCs (*n* = 5). Included genes were not differentially expressed in CNS1 (*n* = 115) compared with HSPCs (*n* = 5). Significance calculated by two-tailed unpaired Students *t* test. **C** Expression pattern of SKAP1 in HSPCs (*n* = 5), CNS1 (*n* = 115), CNS3 (*n* = 10), and CNS relapse (*n* = 13) samples. Data displayed as TPM, mean ± SEM. **D** Expression of SKAP1 in CNS1 (*n* = 115), CNS3 (*n* = 10) and CNS relapse (*n* = 13) samples compared with HSPCs (*n* = 5), separate. Data displayed as TPM, mean ± SEM. Significance calculated by two-tailed unpaired Students *t* test (*P < 0.05). **E** Identification of genes associated with CNS disease relative to both HSPCs and CNS1 T-ALL. A total of three genes were differentially expressed (*P* < 0.05) in both CNS3 (*n* = 10) and CNS relapse (*n* = 13) samples compared with HSPCs (*n* = 5), but not in CNS1 (*n* = 115) compared with HSPCs (*n* = 5), and in CNS3 (*n *= 10) and CNS-relapse (*n* = 13) compared with CNS1 (*n* = 115) samples. **F** Genes (CAPZA2, HINT2, and GLIPR2) significantly altered (*P* < 0.05) in both CNS3 (*n* = 10) and CNS relapse (*n* = 13) compared with both CNS1 (*n* = 115) and HSPCs (*n* = 5). Data displayed as L2FC. Significance calculated by two-tailed unpaired Students *t* test.

To distinguish transcriptional alterations associated with CNS disease from those reflecting general differences between T-ALL cells and hematopoietic cells, we compared CNS1, CNS3, and CNS-relapsed T-ALL samples with cord blood-derived hematopoietic stem and progenitor cells (HSPCs, *n* = 5, obtained from GEO: GSE190269). We first identified genes that were differentially expressed in each T-ALL group compared with HSPCs (Fig. 3A). The majority of differentially expressed genes were shared between the three T-ALL groups (*n* = 5441), most likely representing general transcriptional differences between T-ALL and HSPCs. However, 1573 genes were shared between CNS3 and CNS-relapsed samples but not CNS1 samples, suggesting a transcriptional program associated with CNS disease. Enrichment analysis (ORA) of these CNS-associated genes identified integrin signaling as one of the top enriched KEGG pathways (Figs. 3B and SF3A). Genes enriched within this pathway were ICAM1 and several ITGA family members and laminin subunits, all involved in cell adhesion and ECM interactions (Figs. 1B and SF1B) [26, 27].

We also examined the expression of SKAP1 in both CNS-associated and CNS-negative T-ALL samples compared with HSPCs. SKAP1 expression increased progressively from HSPCs and CNS1 to CNS3 and CNS-relapsed T-ALL samples (Fig. 3C). Although SKAP1 expression was only significantly upregulated in CNS relapse samples, the expression pattern indicates a higher expression in CNS3 and CNS relapse relative to HSPCs and CNS1 samples (Fig. 3D). Similar changes were seen in ITGAL (encodes for LFA1 together with ITGB2), which was upregulated in CNS relapse compared with HSPCs (Fig. SF3C–E). Other components of the inside-out signaling pathway (ITGB2, ADAP1, RAP1A/B, and SKAP2) were downregulated or not significantly altered in all T-ALL groups compared with HSPCs (Fig. SF3C–E). Analysis also identified an upregulation of CDH2, known as N-cadherin, a key regulator of EMT, in both CNS3 and CNS relapse samples, but not in CNS1 samples, compared with HSPCs (Fig. SF3C–E) [28].

Sequentially, we asked whether these CNS-associated genes were also differentially expressed compared with CNS-negative samples. Of the 1 573 genes uniquely shared between CNS3 and CNS relapse samples compared with HSPCs, only three genes were also differentially expressed in CNS3 and CNS relapse compared with CNS1, identified in the earlier analysis (Figs. 1E and 3E). These genes were CAPZA2, GLIPR2, and previously identified HINT2 (Fig. 3F). CAPZA2 has been reported to regulate actin cytoskeleton stability, while GLIPR2 has been associated with EMT and cell migration [29, 30]. Together, these findings identify transcriptional features associated with CNS disease, including genes related to cell adhesion and migration.

### Knockdown of SKAP1 in T-ALL cells reduced cell proliferation and viability

**Fig. 4:**
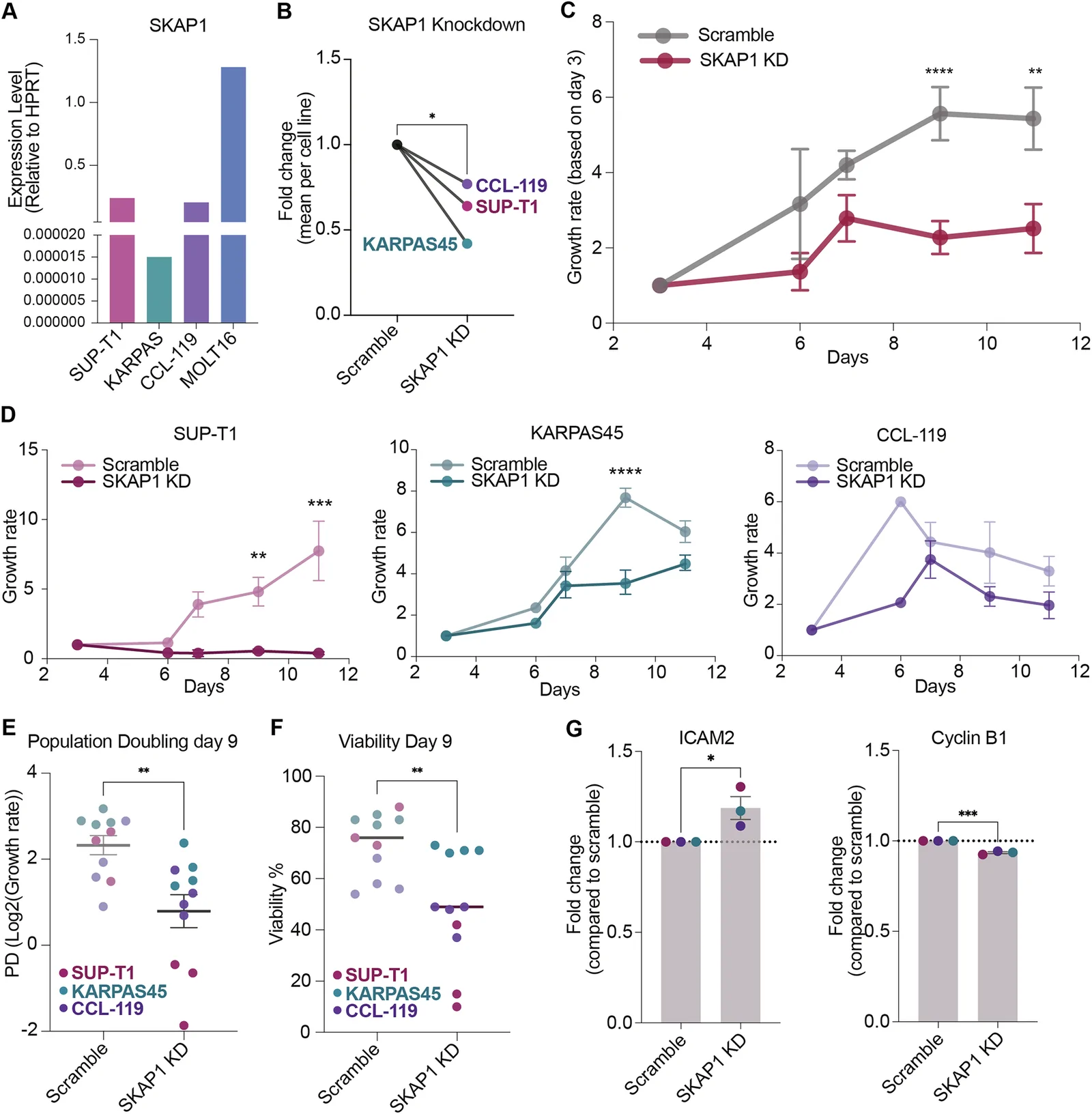
Knockdown of SKAP1 in T-ALL cells reduced cell proliferation and viability. **A** Expression of SKAP1 (relative to HPRT) in T-ALL cell lines SUP-T1, KARPAS45, CCL-119, and MOLT16 (*n* = 1 per cell line). **B** Lentiviral knockdown of SKAP1 compared to scramble control in T-ALL cell lines SUP-T1, KARPAS45, and CCL-119. Data displayed as fold change compared to the scramble control (mean per cell line of replicated experiments). Significance calculated by two-tailed unpaired Students *t* test (*P < 0.05). **C** Growth rate (fold change compared to day 3 post-transduction) of T-ALL cell lines SUP-T1, KARPAS45, CCL-119 (*n* = 3–11 per time-point) from day 3 to day 11 post transduction, in SKAP1 knockdown compared to the scramble control. Significance was calculated using two-way ANOVA with Šídák’s multiple comparisons correction (**P < 0.01, ****P < 0.0001). **D** Growth rate (fold change compared to day 3 post-transduction) of T-ALL cell lines SUP-T1 (*n* = 1–3 per time-point), KARPAS45 (*n* = 1–4 per time-point), CCL-119 (*n* = 1–4 per time-point) separately, from day 3 to day 11 post transduction, in SKAP1 knockdown compared to the scramble control. Significance was calculated using two-way ANOVA with Šídák’s multiple comparisons correction (**P < 0.01, ***P < 0.001, ****P < 0.0001). **E** Population doubling (log2 growth rate based on day 3) on day 9 post-transduction of T-ALL cell lines SUP-T1 (pink, *n* = 3), KARPAS45 (green, *n* = 4), CCL-119 (purple, *n* = 4). Significance calculated by two-tailed unpaired Students *t* test (**P < 0.01). Data displayed as mean ± SEM. **F** Viability (percentage) on day 9 post-transduction of T-ALL cell lines SUP-T1 (pink, *n* = 3), KARPAS45 (green, *n* = 4), CCL-119 (purple, *n* = 4). Significance calculated by two-tailed unpaired Students *t* test (**P < 0.01). Data displayed as mean ± SEM. **G** Expression of ICAM2 and Cyclin B1 (CCNB1) in T-ALL cell lines SUP-T1, KARPAS45, and CCL-119 after SKAP1 knockdown compared to the scramble control. Data displayed as fold change compared to the scramble control (*n* = 3 per condition, cell lines displayed as biological replicates), mean ± SEM. Significance calculated by two-tailed unpaired Students *t* test (*P < 0.05, ***P < 0.001).

To assess the functional effects of SKAP1 and subsequent signaling events, four different T-ALL cell lines (SUP-T1, KARPAS45, CCL-119 and MOLT16), with varying endogenous SKAP1 expression, were utilized for lentiviral knockdown (Fig. 4A). The included T-ALL cell lines displayed different culture patterns, with SUP-T1 and KARPAS45 cells growing in clusters, while CCL-119 and MOL16 cells grew in single-cell suspension (Fig. SF4A). The cells were transduced with shRNA targeting SKAP1 or a scramble control (MOLT16 was excluded due to insufficient knockdown) (Figs. 4B and SF4B). T-ALL cells with diminished SKAP1 expression (cell lines displayed as biological replicates) displayed a significantly lower growth rate compared with the scramble control (Fig. 4C). Notably, cells that grow in clusters under normal conditions (SUP-T1 and KARPAS45) demonstrated a more apparent inhibition of cell growth (Fig. 4D). The cells were observed for up to 11 days post-transduction and the most substantial loss of both population doubling and cell viability was observed at day 9 (Fig. 4E, F). Furthermore, RT-qPCR analysis revealed that knockdown of SKAP1 caused an upregulation of ICAM2 and a modest reduction of cell cycle regulator Cyclin B1 (CCNB1) [31], strengthening our findings regarding the pro-proliferative capacities of SKAP1 in T-ALL (Fig. 4G).

### T-ALL cells exposed to MTX displayed a loss of inside-out signaling when grown in co-culture with HMCs

**Fig. 5:**
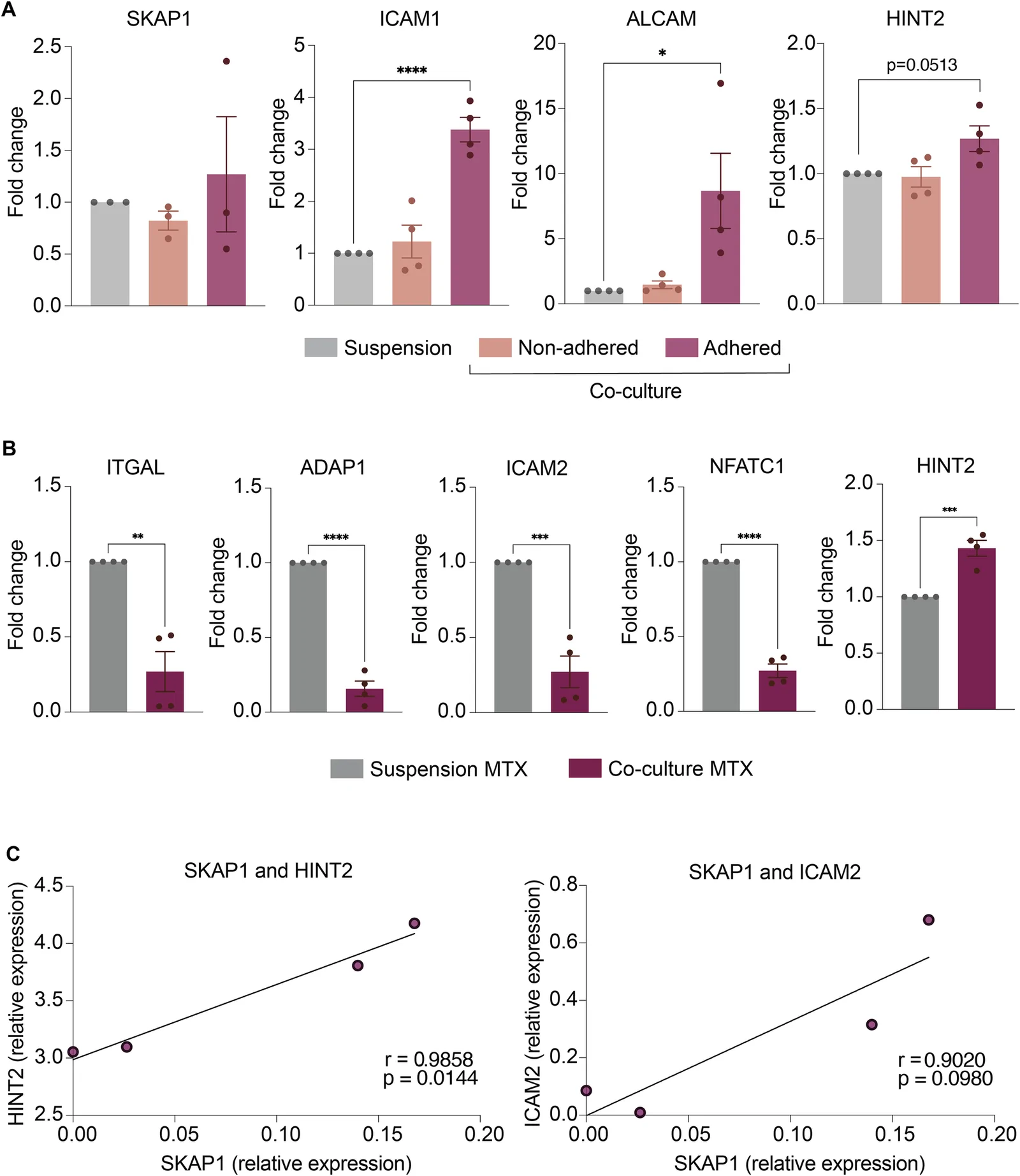
Alterations to genes from the inside-out signaling pathway in MTX-treated T-ALL cells in HMC co-culture. **A** Expression of SKAP1, ICAM1, ALCAM, and HINT2 in T-ALL cell lines SUP-T1, KARPAS45, CCL-119, and MOLT16 grown in HMC co-culture (adhered and non-adhered) compared to T-ALL cells grown alone in suspension. Results displayed as fold change of T-ALL cells (biological replicates *n* = 4 per condition, except for SKAP1 with *n* = 3 due to undetectable expression in KARPAS45) in co-culture compared to their respective control in suspension, mean ± SEM. Significance was calculated using ordinary one-way ANOVA with multiple comparisons compared to suspension, using Dunnett correction (*P < 0.05, ****P < 0.0001). **B** Expression of ITGAL, ADAP1, ICAM2, NFATC1, and HINT2 in T-ALL cell lines SUP-T1, KARPAS45, CCL-119, and MOLT1 grown in HMC co-culture (displayed as biological replicates, *n* = 4 per condition) compared to T-ALL cells grown alone in suspension after exposure to 2 µM MTX. Results displayed as fold change of T-ALL cells in co-culture compared to their respective control in suspension, mean ± SEM. Significance calculated by two-tailed unpaired Students *t* test (**P < 0.01, ***P < 0.001, ****P < 0.0001). **C** Correlation of SKAP1 and HINT2 as well as SKAP1 and ICAM2 in T-ALL cell lines SUP-T1, KARPAS45, CCL-119, and MOLT1 grown in HMC co-culture after exposure to 2 µM MTX (displayed as biological replicates, *n* = 4 per condition). Data displayed as relative expression compared to the housekeeping gene (*x* axis and *y* axis), and results shown as the R value and *P* value of each group. Correlation was calculated using Pearson correlation coefficients, two-tailed with 95% confidence interval.

To analyze effects on inside-out signaling in T-ALL cells interacting with cells from the CNS niche, T-ALL cell lines (SUP-T1, KARPAS45, CCL-119, and MOLT16) were cultured together with CNS-derived human meningeal cells (HMCs) in a co-culture model, or alone in suspension. HMC co-culture did not significantly affect the expression of key components of inside-out signaling, including SKAP1, ADAP, ITGAL, NFATC1, or ICAM2 (Figs. 5A and SF5A). However, the LFA1 ligand ICAM1 and adhesion molecule ALCAM were upregulated in T-ALL cells adhered to HMCs in the co-culture (Fig. 5A). T-ALL cells in co-culture showed an indication of a higher expression of HINT2, which was found to be upregulated in CNS-infiltrated T-ALL samples in the RNA-sequencing analysis (Figs. 1E and 5A).

Next, T-ALL cells were treated with 2 µM MTX, a chemotherapeutic agent routinely used in pediatric T-ALL, including CNS-directed treatment, to assess treatment-induced transcriptomic effects. Previous studies using viability assays have reported increased therapy resistance and survival of T-ALL cells adhered to meningeal cells in co-culture models during MTX exposure [32, 33]. We also assessed viable cell counts following MTX exposure; however, these measurements were not part of a dedicated viability assay and showed substantial variability (Fig. SF5B). When we exposed T-ALL cells to MTX, cells in co-culture underwent changes in expression of several components of the inside-out signaling pathway, including decreased expression of ITGAL and ADAP1, compared to cells in suspension (Fig. 5B). However, no significant alterations in SKAP1 expression were observed (Fig. SF5C). MTX treatment of T-ALL cells led to a downregulation of ICAM2, but not ICAM1 when grown in co-culture (Figs. 5B and SF5C). Cells in MTX-treated co-culture also revealed a downregulation of transcription factor NFATC1 (Fig. 5B). Similar to CNS-infiltrated T-ALL samples from the RNA-sequencing analysis, HINT2 was upregulated in T-ALL cells grown in co-culture (Figs. 1E and 5B). Moreover, a positive correlation was identified between SKAP1 and HINT2, and between SKAP1 and ICAM2 in co-cultured T-ALL cells exposed to MTX (Fig. 5C). These findings indicate that MTX exposure of T-ALL cells in the meningeal co-culture model is associated with transcriptional alterations in genes related to inside-out signaling.

## Discussion

To improve our understanding of the molecular features associated with CNS-infiltration in pediatric T-ALL, we investigated transcriptional alterations in CNS-infiltrated and CNS-relapsed samples. We identified alterations in genes associated with ECM organization, TCR signaling and inside-out signaling, including upregulation of SKAP1. Knocking down SKAP1 led to reduced proliferation and viability of T-ALL cells. Moreover, T-ALL cells co-cultured with meningeal cells displayed loss of components of the inside-out signaling pathway when exposed to MTX. Together, these findings identify TCR- and adhesion-related programs, including SKAP1-associated signaling, as transcriptional features associated with CNS infiltration in pediatric T-ALL.

To predict and prevent CNS infiltration and CNS-originated relapse, we need to understand how leukemia cells invade and persist in the CNS. Increased knowledge on the transcriptional programs associated with CNS infiltration in T-ALL may provide insights into the underlying molecular programs involved in leukemic cell migration. One factor controlling cell migration and metastasis is the ECM, which must be modulated to enable infiltration of cells into other tissues [34–36]. In this study, we identified a transcriptional enrichment of genes involved in ECM organization and degradation, including COL9A3, which has been suggested to regulate blood–CSF permeability through the SOX9-COL9A3 axis, and MMP11, which has been associated with facilitating tumor cell migration [37–39]. Another possible mechanism that could be utilized by leukemia cells is integrin-mediated adhesion, regulated through the TCR and the inside-out signaling pathway, resulting in LFA1-ICAM1 binding [11, 12, 40]. Our study revealed an enrichment of the TCR complex and downstream adapter protein SKAP1 in both CNS-infiltrated and CNS-relapsed T-ALL compared with CNS-negative patients, and SKAP1 upregulation was further validated in a larger T-ALL cohort. In addition, CNS-associated samples exhibited an increased expression of genes implicated in both adhesion and migration, including SKAP1 and N-cadherin (CDH2), compared with HSPCs. These changes were not observed in CNS-negative samples, supporting their association with CNS disease rather than general differences between T-ALL and HSPCs. Together, our results highlight transcriptional alterations in ECM-regulatory genes and several adhesion and migration-associated genes in CNS-infiltrated and CNS-relapsed T-ALL. As these analyses were performed on BM-derived samples, our findings only provide an indirect association with CNS disease, and their functional relevance to leukemic cell migration remains to be established.

We have previously shown that direct interactions between T-ALL cells and HMCs in a CNS co-culture model disrupted cell cycle regulation and induced expression of adhesion molecules [41]. We utilized the same co-culture model to explore inside-out signaling. Although no significant differences were found in SKAP1 expression, an upregulation of ICAM1 was identified in T-ALL cells adhered to meningeal cells. Interestingly, when T-ALL cells were exposed to MTX, a standard component of CNS-directed treatment, there was an overall reduction in components of the inside-out signaling pathway, except for SKAP1, which remained unchanged. Together, these findings indicate that interaction between T-ALL cells and meningeal cells during MTX treatment may be associated with alterations in inside-out signaling.

The specific role of SKAP1 in malignant development remains poorly defined. Overexpression of SKAP1 has been reported in several cancers, including gastric and colon cancer, where it has been associated with tumor progression and immune evasion [42, 43]. Given its prominent role in T-cell biology, aberrant SKAP1 expression may be particularly relevant in T-cell malignancies, such as T-ALL [44]. To address this, we knocked down SKAP1 in T-ALL cell lines, which led to reduced proliferation and viability. Loss of SKAP1 caused a modest downregulation of Cyclin B1, a key regulator of the G2/M transition [31]. Although SKAP1 is not known to directly interact with cyclins or CDKs, it may influence cell-cycle progression through upstream regulators such as PLK1 [45]. Moreover, SKAP1 knockdown in T-ALL cells led to increased ICAM2 expression, which has primarily been recognized for having tumor suppressive effects, but also found to promote migration across the blood–CSF barrier in triple-negative breast cancer [23, 46–48]. While these findings support a role for SKAP1 in T-ALL cell proliferation and viability, our experiments do not establish a role for SKAP1 in leukemic cell migration into the CNS. Dedicated migration assays and in vivo models will be required to determine the functional significance.

Our findings point to SKAP1 and inside-out signaling as part of transcriptional programs associated with cell proliferation and an indirect link to CNS infiltration in pediatric T-ALL. Canonically, inside-out signaling is initiated downstream of the TCR [12]. However, T-ALL arises from thymocytes arrested at distinct developmental stages, resulting in heterogeneous CD3 and TCR surface expression [49]. While some T-ALL subtypes express a fully assembled αβ TCR, others express an incompletely assembled pre-TCR or lack surface TCR expression altogether [50]. This heterogeneity complicates the interpretation of TCR-dependent signaling in T-ALL. Notably, we observed increased SKAP1 expression in CNS3 and CNS-relapsed T-ALL without directly assessing TCR activation. Whether SKAP1-associated signaling in T-ALL depends on TCR engagement or can be regulated through alternative pathways therefore remains an open question that warrants further functional investigation.

A limitation of this study is the use of BM-derived samples, which may not fully capture the molecular landscape of leukemia cells within the CNS microenvironment. However, given the challenges associated with obtaining CSF samples from pediatric patients, BM represents a practical and clinically relevant source of material. Importantly, BM-derived leukemia samples retained transcriptional features associated with CNS disease, distinguishing CNS-infiltrated and CNS-relapsed samples from CNS-negative samples. These findings support the use of BM samples for identifying transcriptional features associated with CNS disease, but future studies integrating matched BM and CSF samples will be important to further validate these findings.

In conclusion, our findings identify SKAP1 and altered TCR- and adhesion-related signaling as transcriptional features associated with CNS disease in pediatric T-ALL. These findings provide new insight into transcriptional programs associated with CNS infiltration in T-ALL and highlight molecular processes that warrant further functional investigation.

## Methods

### Downloading and normalization of publicly available RNA-sequencing data

RNA-sequencing data from pediatric T-ALL patients (*n* = 180) were publicly available by the TARGET initiative (phs000218). The RNA-sequencing dataset (.BAM) was acquired via the GDC Data Portal (https://portal.gdc.cancer.gov/projects/TARGET-ALL-P2). A total of 180 BM-derived T-ALL samples were included, 162 from the time of diagnosis and 18 from relapsed patients (not longitudinal). Diagnosis samples were subdivided by the clinical characterization for CNS infiltration, into CNS1 (*n* = 115), CNS2 (*n* = 37), or CNS3 (*n* = 10) [15]. Relapse samples were grouped depending on the site of relapse, either BM (*n* = 5) or CNS (*n* = 13). Raw counts were generated using the SeqMonk Mapped Sequence Data Analyzer tool (v1.47.1) with the pipeline described in Enlund et al. [41]. RNA-sequencing from CD34^+^ cord blood was obtained from a publicly available dataset (GSE190269) from the Gene Expression Omnibus [51]. Raw counts were normalized in R (v4.3.1) to transcripts per million (TPM) following the GDC bioinformatics pipeline. Non-coding and novel genes were excluded from the analysis. The pediatric T-ALL validation cohort (Kids First, phs002276) was obtained from Synapse (syn54032669). TPM expression was downloaded for 799 T-ALL samples, from diagnosis (*n *= 716) and relapse (*n* = 83), with a total of 724 CNS1 and 75 CNS3 samples [22].

### Functional enrichment analysis

Functional gene set enrichment analysis and ORA were performed using R (v4.3.1) with the packages EnrichR (v3.2), clusterProfiler (v4.8.3), and enrichplot (v1.20.3). EnrichR analysis included common differentially expressed genes in CNS3 (*n* = 10) and CNS relapse (*n* = 123) vs CNS1 (*n* = 115) samples. ORA included all differentially expressed genes with a L2FC above 2 in CNS3 and CNS relapse vs CNS1 samples, respectively.

### Cell culture

T-ALL cell lines CCL-119 and SUP-T1 (ATCC, Manassas, VA, USA) were cultured in RPMI (Sigma-Aldrich, St. Louis, MO, USA) supplemented with 10% FBS (Sigma-Aldrich), MOLT16 (Leibniz Institute DSMZ, Braunschweig, Germany) was cultured in IMDM (Gibco, Thermo Fisher Scientific, Grand Island, NY, USA) supplemented with 10% FBS, and KARPAS-45 was cultured in RPMI (Sigma-Aldrich), supplemented with 2 mM glutamine and 20% FBS (Sigma-Aldrich). Immortalized Human Meningeal Cells (IM-HMCs) (Innoprot, Bizkaia, Spain) were cultured in complete Meningeal Cell Medium (Innoprot) and detached using the Primary Cells Detach Kit (Innoprot).

### Reverse transcription and RT-qPCR

RNA preparation (Qiagen, Hilden, Germany), reverse transcription (Invitrogen, Carlsbad, CA, USA) and RT-qPCR analysis (Invitrogen) were performed according to the provided protocols and as described in Enlund et al. [41]. Primer sequences can be found in the Supplementary Table S1.

### Lentiviral knockdown

Human shRNA lentiviral particles targeting SKAP1 and a scramble control (TL309635V, OriGene Technologies, Rockville, MD, USA) were used to knock down SKAP1 in T-ALL cell lines (each cell line represents a biological replicate, *n* = 4 per condition, with 3–4 technical replicates per cell line). Lentiviral transduction was performed at a multiplicity of infection (MOI) of 20 to 50, and cells were harvested 72 h post-transduction. Viability and live-cell count were quantified by trypan blue staining (Bio-Rad, Hercules, CA, USA), and knockdown efficacy was determined by RT-qPCR.

### Co-culture

To determine the effects of interactions between T-ALL cells and meningeal cells, we utilized a previously established CNS co-culture model, as described in Enlund et al. [41]. T-ALL cell lines were cultured either alone in suspension or together with IM-HMCs (Innoprot) at a ratio of 3:1 leukemia cells to IM-HMCs (each cell line represents a biological replicate, *n* = 4 per condition). Non-adherent cells were collected after 24 h followed and adhered cells were harvested and separated from IM-HMCs using EasySep Human CD3-positive Selection kit II (Stem Cell Technologies) after an additional 48 h. In conditions where cells were treated with chemotherapy, 2 µM of MTX (Sigma-Aldrich) was added after 24 h. Viability and cell count were determined using trypan blue staining (Bio-Rad).

### Statistical analysis

Statistical analyses were performed using unpaired two-tailed Students *t* test, one-way ANOVA with Dunnett’s correction, or two-way ANOVA with Šídák’s multiple comparisons correction. Correlation was determined by Pearson correlation coefficients, two-tailed with 95% confidence interval. Analyses were performed using GraphPad Prism (v10) or R (version 4.3.1). Results are presented as the mean ± SEM. Significance was set to *P* value < 0.05.

## Supplementary information

Supplementary information

Supplemental table 1

Supplemental table 2

Supplemental table 3

Supplemental table 4

## Data Availability

The RNA-sequencing data analyzed in this study were obtained from publicly available datasets. The main T-ALL dataset used in this study is based upon data generated by the Therapeutically Applicable Research to Generate Effective Treatments (TARGET) initiative, phs000218, managed by the NCI. The data used for this analysis are available at https://portal.gdc.cancer.gov/projects/TARGET-ALL-P2. The validation T-ALL cohort was generated through Kids First (phs002276) and obtained from Synapse (syn54032669). RNA-sequencing data from HSPCs were obtained from GEO (GSE190269). Further information and requests for resources and reagents should be directed to and will be fulfilled by Dr. Frida Holm (frida.holm@ki.se).
