# Supplementary material for: Transcriptional alterations in SKAP1-associated inside-out signaling in CNS-infiltrated pediatric T-cell acute lymphoblastic leukemia
Source: Cell Death Discov. 2026 Nov 5;12:443. doi: 10.1038/s41420-026-03452-w (PMC13740306; doi:10.1038/s41420-026-03452-w)
Supplement: Supplementary file 1 [file 41420_2026_3452_MOESM1_ESM.pdf]

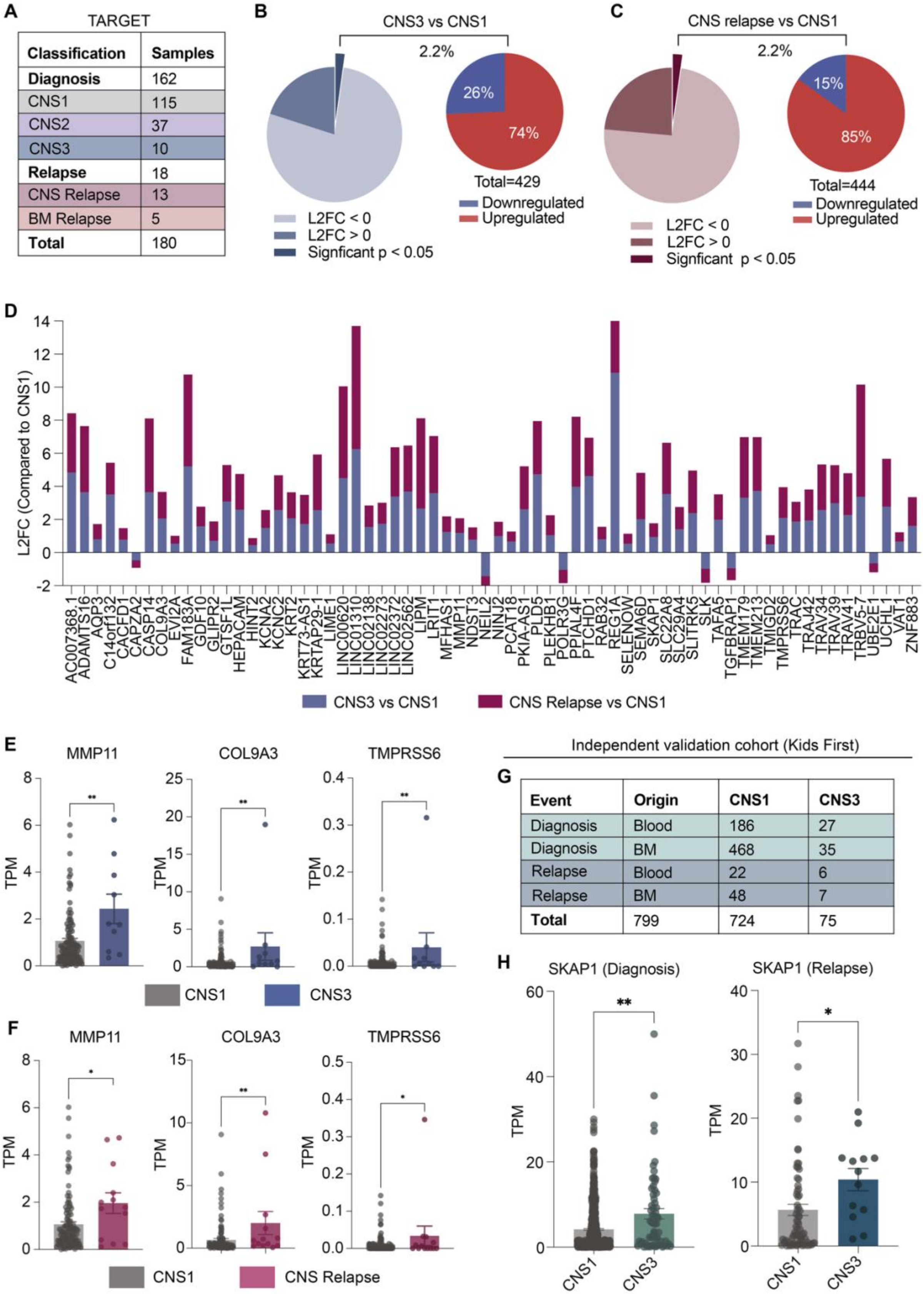

Classification	Samples
**Diagnosis**	162
CNS1	115
CNS2	37
CNS3	10
**Relapse**	18
CNS Relapse	13
BM Relapse	5
**Total**	180

Event	Origin	CNS1	CNS3
Diagnosis	Blood	186	27
Diagnosis	BM	468	35
Relapse	Blood	22	6
Relapse	BM	48	7
**Total**	799	724	75

Supplemental figure 1.

A) Table of patient characteristics of pediatric T-ALL samples (total n=180). Samples from time of diagnosis are subdivided based on the clinical characterization of CNS infiltration, into CNS1 (n=115), CNS2 (n=37) or CNS3 (n=10). Samples taken at relapse were subdivided by site of relapse; CNS (n=13) or BM (n=5).

B) Distribution of all genes in CNS3 (n=10) compared to CNS1 (n=115) samples, defined by if the L2FC is above or below 0, as well as all significantly expressed genes (2.2%, $p<0.05$). Distribution of all significantly expressed genes (n=429), with 74% upregulated genes (L2FC>0) and 26% downregulated (L2FC<0). Significance calculated by two-tailed unpaired students t-test.

C) Distribution of all genes in CNS relapse (n=13) compared to CNS1 (n=115) samples, defined by if the L2FC is above or below 0, as well as all significantly expressed genes (2.2%, $p<0.05$). Distribution of all significantly expressed genes (n=444), with 85% upregulated genes (L2FC>0) and 15% downregulated (L2FC<0). Significance calculated by two-tailed unpaired students t-test.

D) Common differentially expressed genes ($p<0.05$, total of 66 genes) in CNS3 (n=10) and CNS relapse (n=13) compared to CNS1 (n=115). Data displayed as L2FC. Significance calculated by two-tailed unpaired students t-test.

E) Expression of MMP11, COL9A3 and TMPRSS6 in CNS3 (n=10) compared to CNS1 (n=115) samples. Data displayed as TPM, mean ± SEM. Significance calculated by two-tailed unpaired students t-test (*$p<0.05$, **$p<0.01$).

F) Expression levels of MMP11, COL9A3 and TMPRSS6 in CNS relapse (n=13) samples compared to CNS1 (n=115) samples. Data displayed as TPM, mean ± SEM. Significance calculated by two-tailed unpaired students t-test (*$p<0.05$, **$p<0.01$).

G) Table of patient characteristics of pediatric T-ALL samples from the Kids First cohort (n=799), grouped by sample source, CNS status and event stage.

H) Expression of SKAP1 in CNS3 compared to CNS1 samples in diagnosis (CNS1 n=654, CNS3 n=62) and relapse (CNS1 n=70, CNS3 n=13) separate, from the Kid's First dataset. Data displayed as TPM, mean ± SEM. Significance calculated by two-tailed unpaired students t-test (*$p<0.05$, **$p<0.01$)

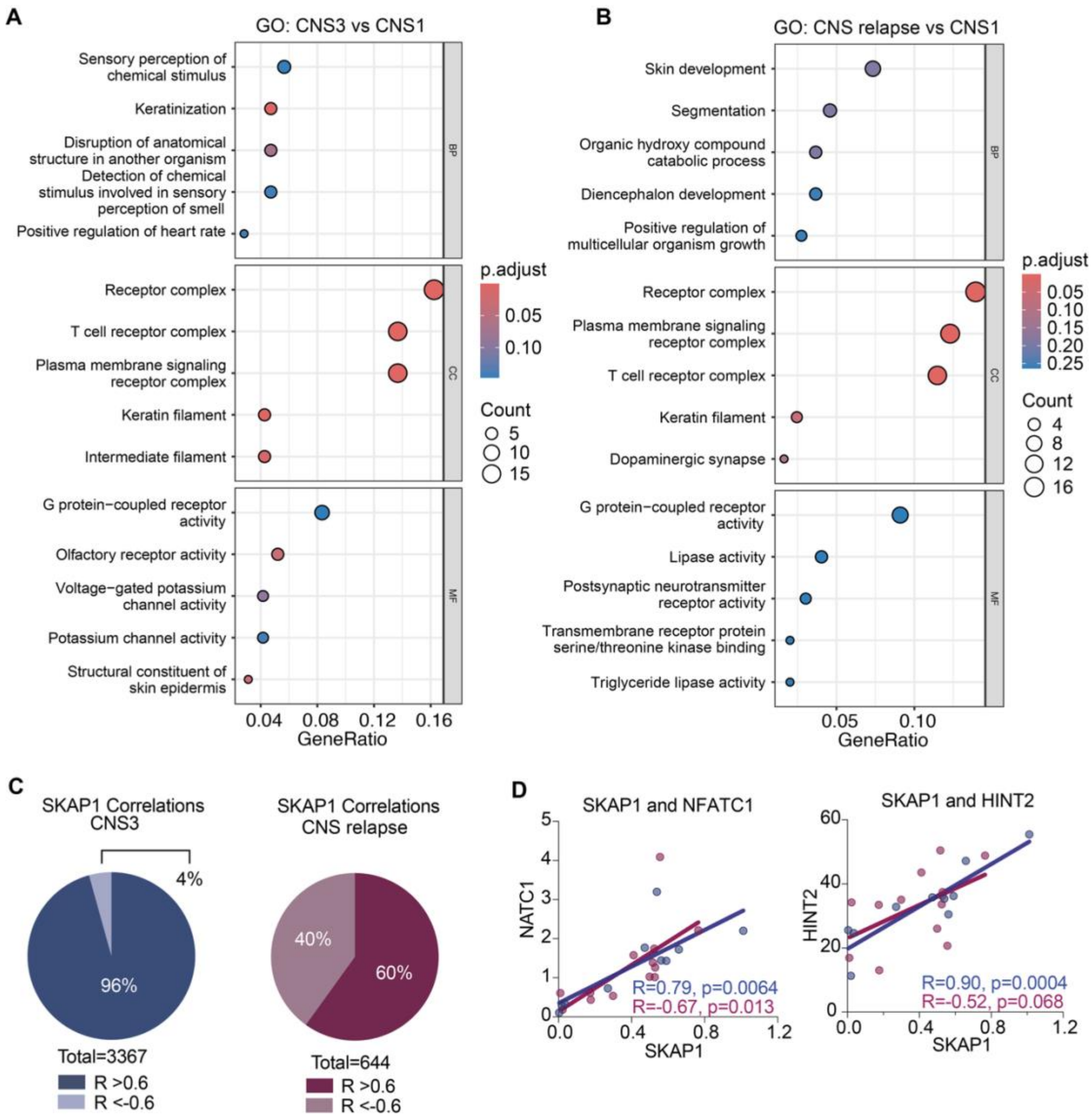

Supplemental figure 2.

A) ORA of differentially expressed genes (p<0.05, L2FC>2) in CNS3 (n=10) compared to CNS1 (n=115) samples, using GO gene sets, showing biological processes (BP), cellular component (CC) and molecular function (MF). Significance displayed as adjusted p-value.

B) ORA of differentially expressed genes (p<0.05, L2FC>2) in CNS relapse (n=13) compared to CNS1 (n=115) samples, using gene ontology gene sets, showing biological processes (BP), cellular component (CC) and molecular function (MF). Significance displayed as adjusted p-value.

C) Distribution of SKAP1 correlations with other genes in CNS3 samples (n=10, left graph) and CNS relapse samples (n=13, right graph), with an R value above 0.6 or below -0.6. Correlation was calculated using Pearson correlation coefficients, two-tailed with 95% confidence interval.

D) Correlations of SKAP1 and NFATC1 and HINT2 in CNS3 (n=10) and CNS relapse (n=13) samples separately (CNS3 in blue, CNS relapse in maroon). Correlation was calculated using Pearson correlation coefficients, two-tailed with 95% confidence interval. Data displayed as TPM values (x-axis and y-axis) and results shown as the R value and p-value of each group.

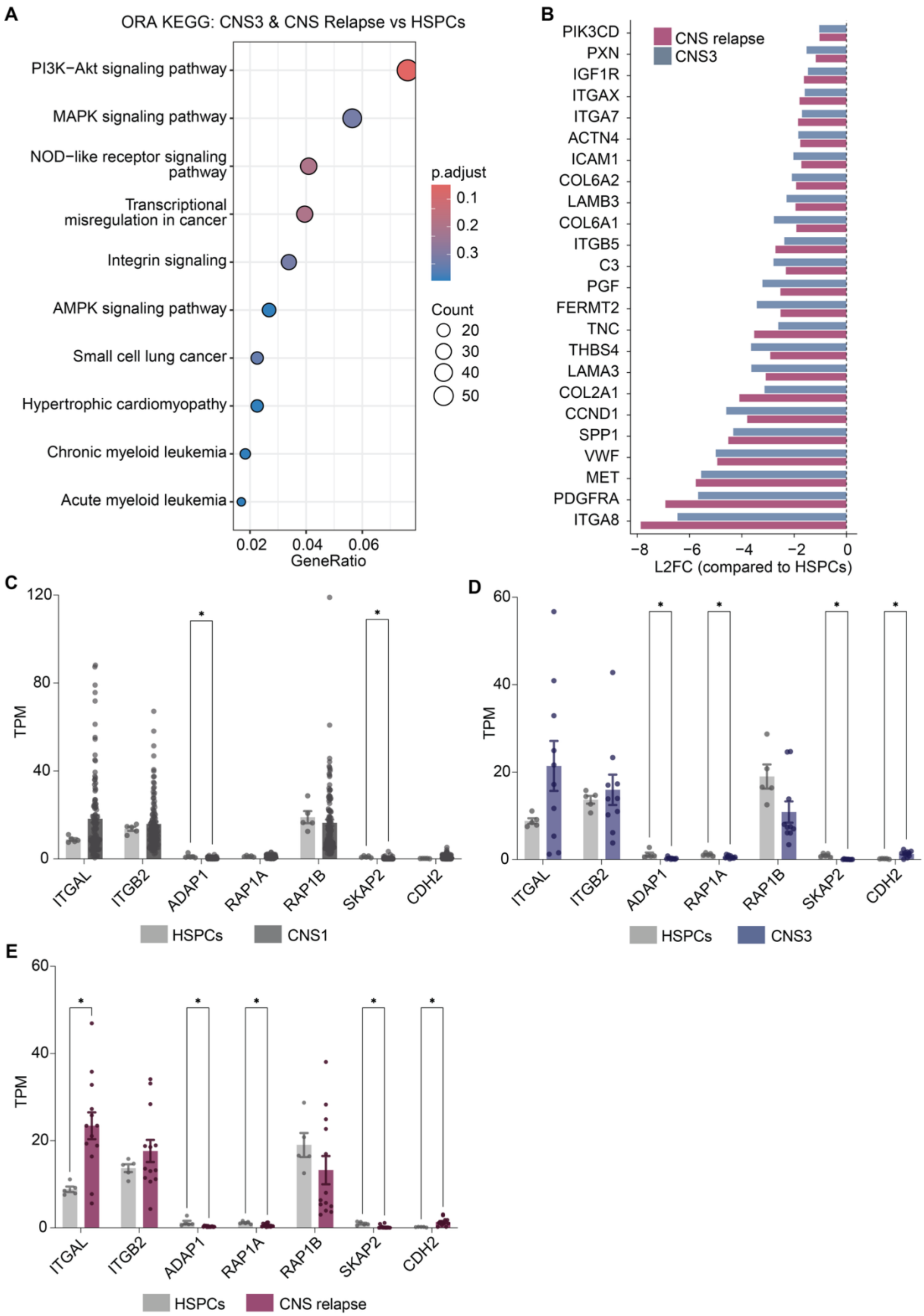

A
ORA KEGG: CNS3 & CNS Relapse vs HSPCs
PI3K−Akt signaling pathway
MAPK signaling pathway
NOD−like receptor signaling pathway
Transcriptional misregulation in cancer
Integrin signaling
AMPK signaling pathway
Small cell lung cancer
Hypertrophic cardiomyopathy
Chronic myeloid leukemia
Acute myeloid leukemia
0.02
0.04
0.06
GeneRatio
p.adjust
0.1
0.2
0.3
Count
20
30
40
50
B
CNS relapse
CNS3
PIK3CD
PXN
IGF1R
ITGAX
ITGA7
ACTN4
ICAM1
COL6A2
LAMB3
COL6A1
ITGB5
C3
PGF
FERMT2
TNC
THBS4
LAMA3
COL2A1
CCND1
SPP1
VWF
MET
PDGFRA
ITGA8
−8
−6
−4
−2
0
L2FC (compared to HSPCs)
C
TPM
120
80
40
0
ITGAL
ITGB2
ADAP1
RAP1A
RAP1B
SKAP2
CDH2
HSPCs
CNS1
D
TPM
60
40
20
0
ITGAL
ITGB2
ADAP1
RAP1A
RAP1B
SKAP2
CDH2
HSPCs
CNS3
E
TPM
60
40
20
0
ITGAL
ITGB2
ADAP1
RAP1A
RAP1B
SKAP2
CDH2
HSPCs
CNS relapse

Supplemental figure 3.

A) Top enriched KEGG pathways in CNS3 (n=10) and CNS relapse (n=13) compared to HSPCs (n=5), based on differentially expressed genes ($p<0.05$) exclusive to CNS3 and CNS relapse compared to HSPCs. Included genes were not differentially expressed in CNS1 (n=115) compared with HSPCs. Significance displayed as adjusted p-value.

B) Enriched genes ($p<0.05$) belonging to the KEGG pathway Integrin signaling, exclusive to CNS3 (n=10) and CNS relapse (n=13) compared with HSPCs (n=5). Included genes were not differentially expressed in CNS1 (n=115) compared with HSPCs (n=5). Data is displayed as L2FC compared with HSPCs. Significance calculated by two-tailed unpaired students t-test.

C) Expression of ITGAL, ITGB2, ADAP1, RAP1A, RAP1B, SKAP2, and CDH2 in CNS1 samples (n=115) compared with HSPCs (n=5). Data displayed as TPM, mean ± SEM. Significance calculated by multiple two-tailed, unpaired student's t-test with Holm-Šídák correction for multiple comparisons, displayed as adjusted p-value (*$p<0.05$).

D) Expression of ITGAL, ITGB2, ADAP1, RAP1A, RAP1B, SKAP2 and CDH2 in CNS3 samples (n=10) compared with HSPCs (n=5). Data displayed as TPM, mean ± SEM. Significance calculated by multiple two-tailed, unpaired student's t-test with Holm-Šídák correction for multiple comparisons, displayed as adjusted p-value (*$p<0.05$).

E) Expression of ITGAL, ITGB2, ADAP1, RAP1A, RAP1B, SKAP2 and CDH2 in CNS relapse samples (n=13) compared with HSPCs (n=5). Data displayed as TPM, mean ± SEM. Significance calculated by multiple two-tailed, unpaired student's t-test with Holm-Šídák correction for multiple comparisons, displayed as adjusted p-value (*$p<0.05$).

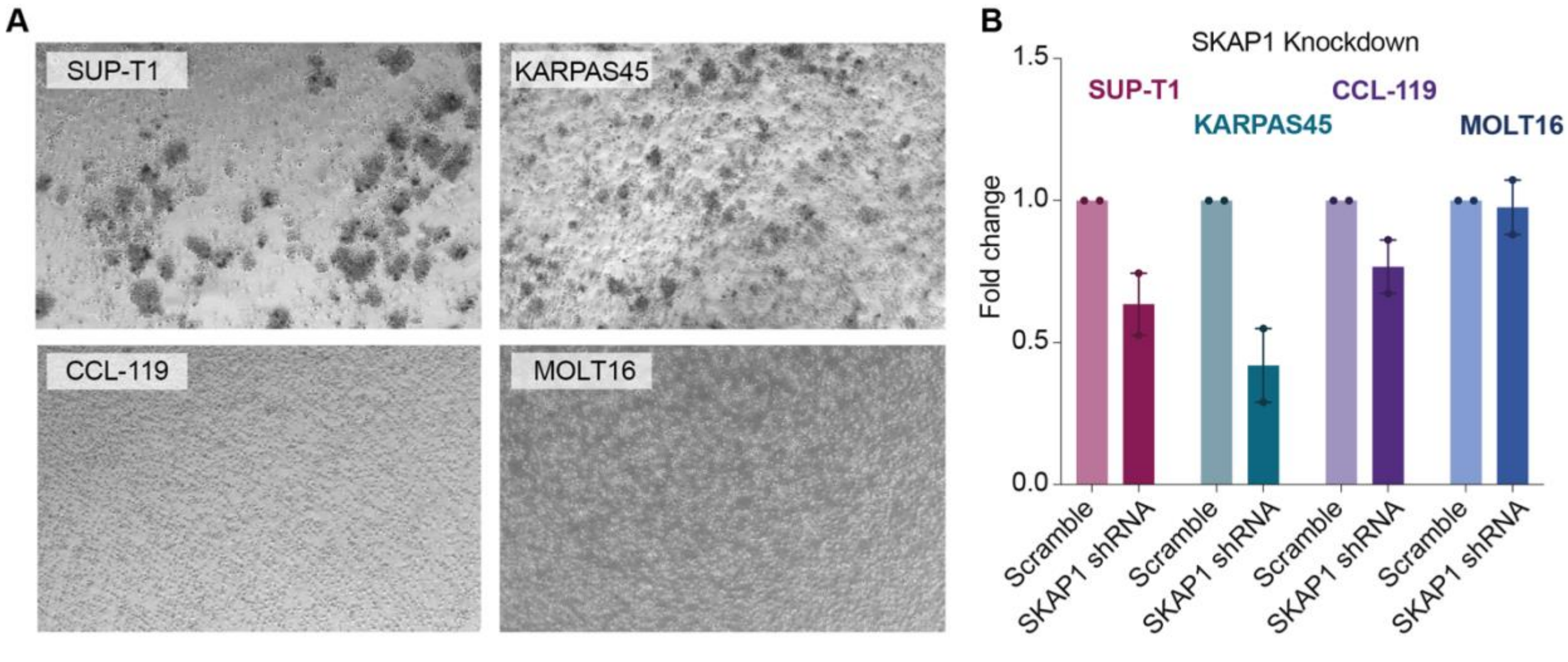

Supplemental figure 4.

A) Representative brightfield microscopy images of the T-ALL cell lines SUP-T1, KARPAS45, CCL-119 and MOLT16. Images were acquired using a 4× objective.

B) Lentiviral knockdown of SKAP1 compared to scramble control in T-ALL cell lines SUP-T1, KARPAS45, CCL-119 and MOLT16. Data displayed as fold change compared to the scramble control (two replicates per cell line), mean ± SEM.

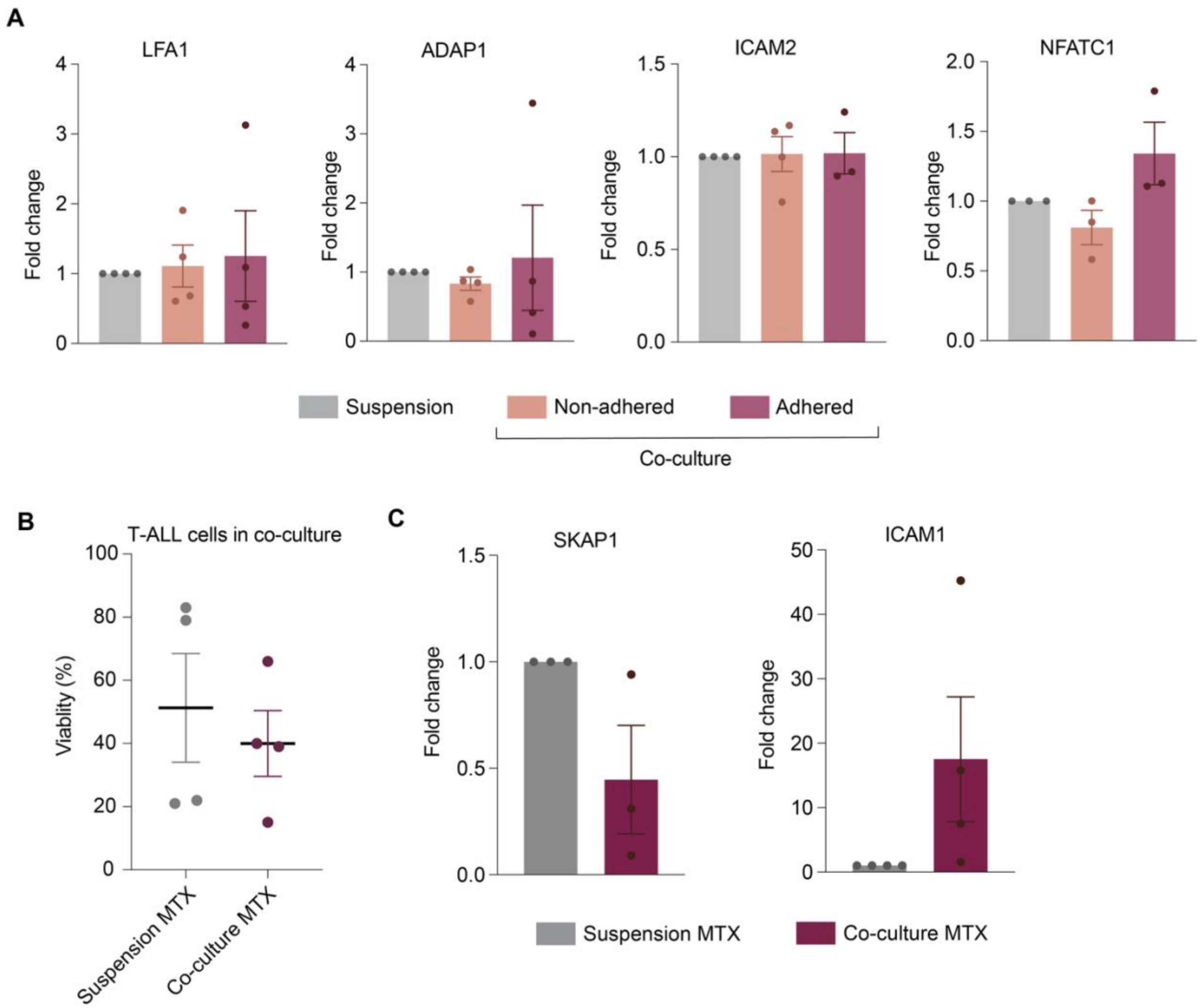

Supplemental figure 5.

A) Expression of ITGAL, ADAP1, ICAM2 and NFATC1 in T-ALL cell lines SUP-T1, KARPAS45, CCL-119 and MOLT16 grown in HMC co-culture (adhered and non-adhered) compared to T-ALL cells grown alone in suspension. Results displayed as fold change of T-ALL cells (displayed as biological replicates, n=4 per condition) in co-culture compared to their respective control in suspension, mean ± SEM. Significance was calculated using ordinary one-way ANOVA with multiple comparisons compared to suspension, using Dunnett correction.

B) Viable cell count (percent viable cells) in T-ALL cell lines SUP-T1, KARPAS45, CCL-119 and MOLT16 exposed to 2uM MTX in suspension or in co-culture with HMCs (biological replicates, n=4 per condition). Data displayed as mean ± SEM

C) Expression of ICAM1 and SKAP1 in T-ALL cell lines SUP-T1, KARPAS45, CCL-119 and MOLT16 grown in HMC co-culture (displayed as biological replicates, n=4 per condition)

compared to T-ALL cells grown alone in suspension after exposure to 2uM MTX. Results displayed as fold change of T-ALL cells in co-culture compared to their respective control in suspension, mean ± SEM. Significance calculated by two-tailed unpaired students t-test.

Supplemental Tables

Supplemental Table 1. Differentially expressed genes in T-ALL; CNS3 vs CNS1 (tab 1) and CNS relapse vs CNS1 (tab 2).

Supplemental Table 2. Expression of SKAP1 in the Kids Frist T-ALL cohort.

Supplemental Table 3. Differentially expressed genes in T-ALL compared to HSPCs; CNS1 vs HSPCs (tab 1), CNS3 vs HSCPs (tab 2) and CNS relapse vs HSPCs (tab 3).

Supplemental Table 4. Primer sequences.
